# Nutrients, Phytochemicals, and In Vitro Biological Activities of Goldenberry (*Physalis peruviana* L.) Fruit and Calyx

**DOI:** 10.3390/plants14030327

**Published:** 2025-01-22

**Authors:** Mikel Añibarro-Ortega, Maria Inês Dias, Jovana Petrović, Filipa Mandim, Sonia Núñez, Marina Soković, Víctor López, Lillian Barros, José Pinela

**Affiliations:** 1 CIMO, LA SusTEC, Instituto Politécnico de Bragança, Campus de Santa Apolónia, 5300-253 Bragança, Portugal; 2 Nutrition and Bromatology Group, Department of Analytical and Food Chemistry, Faculty of Food Science and Technology, University of Vigo, Ourense Campus, 32004 Ourense, Spain; 3 Institute for Biological Research “Siniša Stanković”, National Institute of Republic of Serbia, University of Belgrade, Bulevar despota Stefana 142, 11108 Belgrade, Serbia; 4 Department of Pharmacy, Faculty of Health Sciences, Universidad San Jorge, Villanueva de Gállego, 50830 Zaragoza, Spain; 5 Instituto Agroalimentario de Aragón, IA2, Universidad de Zaragoza-CITA, 50830 Zaragoza, Spain; 6 National Institute for Agricultural and Veterinary Research (INIAV), I.P., Rua dos Lágidos, Lugar da Madalena, Vairão, 4485-655 Vila do Conde, Portugal

**Keywords:** nutritional composition, polyphenols, antioxidant activity, enzyme inhibition capacity, antidiabetic potential, anti-inflammatory potential, antimicrobial activity, antiproliferative activity

## Abstract

This study provides a comprehensive characterization of *Physalis peruviana* L., covering the nutritional composition of the fruit and the phytochemical profiles and in vitro bioactive properties of berry and calyx extracts. The fresh fruit stood out as a source of dietary fiber (5.16 g/100 g) and is low in fat (0.49 g/100 g). A 100-g serving also contained notable amounts of ascorbic acid (32.0 mg), tocopherols (2.34 mg), potassium (253 mg), phosphorus (45 mg), and magnesium (20 mg). HPLC-DAD-ESI/MS analysis tentatively identified five physalin derivatives and one withanolide in the fruit extract, which showed significant antiproliferative activity against human colorectal adenocarcinoma (Caco-2) and non-small-cell lung carcinoma (NCI-H460) cells. The calyx extracts contained three phenolic acids and four flavonoids, demonstrating high antioxidant activity through physiologically relevant cell-based assays, the ability to inhibit advanced glycation end products (AGEs) formation and nitric oxide production, and also antiproliferative properties. These findings highlight goldenberry as a nutrient-dense fruit rich in vitamins and functional compounds with potential health benefits, supporting its recognition as a “superfruit”. Furthermore, the fruit calyx emerged as a valuable source of bioactive secondary metabolites with potential applications in food and pharmaceutical industries and related sectors.

## 1. Introduction

The growing consumer interest in exotic fruits, driven by their nutritional value, health benefits, and unique organoleptic characteristics, has led to the commercialization and cultivation of tropical fruits in various European countries [1]. Among these fruits, *Physalis peruviana* L., commonly known as goldenberry or cape gooseberry, has emerged and gained recognition as a functional food or “superfruit” due to its health-promoting effects and exquisite citrus flavor [2]. Native to the Peruvian and Ecuadorian Andes, this herbaceous, semi-shrub perennial plant (in subtropical zones) produces orange-yellow berries approximately 1.25–2 cm in diameter. These berries contain juicy pulp with 100–300 very small yellowish seeds and are encased in an inflated papery calyx, which accounts for about 5% of the fresh fruit’s weight [3]. Introduced to South Africa by the Spanish, the species has spread to tropical and subtropical regions and is now cultivated in Central and Southern Europe, the United States, Asia, and the Pacific [2].

Goldenberry is regarded as a valuable commodity, consumed not only fresh but also in the dried form and processed into various products such as jam, juice, syrup, and appetizers [4], as well as in freeze-dried powder formulations [5]. During industrial processing, the fruit calyx is typically discharged as a by-product with no commercial value [6,7]. However, *P. peruviana* calyx has been studied for its ability to modulate glucose metabolism in both in vitro and in vivo settings [8]. Research has highlighted the antioxidant and antiproliferative properties of goldenberry fruit and calyx extracts, which are attributed to their content of phenolic compounds, terpenes, polysaccharides, and steroidal compounds such as physalins and withanolides [9,10,11,12,13]. Furthermore, it was demonstrated that regular consumption of goldenberry prevents insulin resistance and obesity in rats, emphasizing its health benefits and protective role against metabolic syndrome [14].

Oxidative stress is characterized by an imbalance between the production of reactive oxygen and nitrogen species and the body’s ability to detoxify these reactive products or repair the resulting damage. This imbalance can lead to tissue damage and is implicated in various diseases and aging processes [15,16]. The inhibition of oxidative stress has gained attention due to its connection with conditions such as atherosclerosis, cardiovascular disease, cancer, and neurodegenerative disorders [17]. Many herbal preparations commonly used in folk medicine exhibit antioxidant properties, primarily due to bioactive constituents such as phenolic compounds [18]. Previous studies have attributed antioxidant activity to fruit and calyx extracts through chemical assays, primarily based on free radical scavenging reactions [7,11]. However, these methods often fail to mimic physiological conditions and rely on radicals (e.g., DPPH and ABTS) that are not naturally present in the human body. Additionally, some studies have reported hepatoprotective and nephroprotective properties for the calyx [19,20], while both the fruit and calyx have been suggested to ameliorate symptoms related to metabolic syndrome, including diabetes and obesity [8,21,22,23]. Despite these findings, the literature on the effectiveness of *P. peruviana* fruit and calyx extracts in addressing oxidative stress-mediated conditions, such as inflammation, diabetes, obesity, and cancer, remains limited and warrants further investigation. Consequently, there is a need for in-depth studies to investigate the compositional and bioactive features of this berry and its calyx. Such research could support their sustainable incorporation into the development of novel food, nutraceutical, and pharmaceutical products.

Given the abovementioned considerations, this study aimed to investigate the compositional and bioactive characteristics of the *P. peruviana* fruit and calyx produced in the Northeast region of Portugal, a non-native area with distinct edaphoclimatic conditions. Specifically, the nutritional composition (centesimal composition, mineral elements, and individual hydrophilic and lipophilic constituents) of the fruit was characterized, along with the phytochemical profiles and in vitro bioactive properties of both fruit and calyx extracts, including antioxidant, anti-inflammatory, antidiabetic, anti-obesity, antimicrobial, and cytotoxic effects. In addition to hydroethanolic extracts, a decoction of the calyx was prepared following traditional folk medicine practices to further explore this lesser-known herbal preparation. The goal was to provide comprehensive insights into this promising “superfruit” and highlight its potential applications across the food and related industries, while also promoting the valorization of the underutilized calyx.

## 2. Materials and Methods

### 2.1. Plant Material, Chemicals, and Standards

Goldenberries grown in Carrazeda de Ansiães, a municipality of Bragança, Portugal, were purchased (1 kg) from a local market in September 2019 at their commercial maturity stage. The fruit (which accounted for 96.5% of the total weight) was separated from the dry calyx (which comprised the remaining 3.5%) and analyzed for moisture content using a PMB moisture analyzer (Adam Equipment, Kingston, Milton Keynes, UK). The fresh whole fruits were freeze-dried (VaCo 2, Zirbus Technology, Bad Grund, Germany) until a constant weight was achieved. Both samples (fruit and calyx) were finely ground into a homogenous powder of approximately 20 mesh particle size using a domestic grinder and then vacuum-packaged and stored at −20 °C for further analysis. The moisture content of the freeze-dried fruit powder was determined.

The chemicals, standards, and biological materials used in this study are listed in Table S1.

### 2.2. Nutritional Analysis of the Fruit

#### 2.2.1. Proximate Composition and Energy

The fruit sample was analyzed for protein, fat, ash, and total dietary fiber contents following official AOAC methods [24]. Protein (N × 6.25) was estimated by the macro-Kjeldahl method (AOAC 920.152), crude fat by Soxhlet extraction with petroleum ether (AOAC 920.85), ash by incineration in a muffle furnace (AOAC 940.26), and total dietary fiber via an enzymatic–gravimetric method (AOAC 985.29). Available carbohydrates were estimated by weight difference. The results were given as g/100 g of plant material on a fresh (fw) and dry (dw) weight basis. The energy value (kcal/100 g fw and dw) was calculated considering the conversion factors: 9 kcal/g for fat, 4 kcal/g for protein and available carbohydrates, and 2 kcal/g for fiber (Regulation (EU) No 1169/2011) [25].

#### 2.2.2. Mineral Elements

Mineral elements were analyzed by atomic absorption spectroscopy (AAS) using a Perkin Elmer PinAAcle 900T Spectrometer (Waltham, MA, USA). The powdered berry sample was digested with nitric acid and analyzed as described by Othman et al. [26]. Before AAS analysis, specific treatments were applied as follows: for potassium (K) and sodium (Na), the solution was diluted in a cesium chloride solution; for calcium (Ca) and magnesium (Mg), it was diluted in a lanthanum chloride solution; and manganese (Mn), copper (Cu), iron (Fe), and zinc (Zn) were directly analyzed. Phosphorus (P) was analyzed using a colorimetric method [27]. Quantification (mg/100 g fw and dw) was achieved by comparing the sample response with that of standard analytical solutions.

#### 2.2.3. Hydrophilic Constituents

Free sugars were analyzed using a Knauer high-performance liquid chromatography (HPLC) system as described by Pinela et al. [28]. The powdered berry sample was mixed with melezitose (25 mg/mL) and subjected to solid-liquid extraction with 80% ethanol at 80 °C for 90 min. After filtration, the supernatant underwent concentration, and the residue was dissolved in distilled water and filtered through a 0.2 µm filter disk. Chromatographic separation was conducted on a Knauer Eurospher 100-5 NH_2_ column (5 mm, 4.6 × 250 mm) using acetonitrile/water (70:30, *v*/*v*) as mobile phase and the signal was recorded with a Smartline 2300 refraction index detector. Free sugars were identified by chromatographic comparisons with standard compounds from Sigma-Aldrich (St. Louis, MO, USA) and quantified (g/100 g fw and dw) using the internal standard method.

Organic acids were analyzed using a Shimadzu LC-20A series HPLC system, following a methodology formerly described [29]. The powdered berry sample was stirred with meta-phosphoric acid for 45 min and filtered through a Whatman no. 4 filter paper and a 0.2 µm filter disk. Chromatographic separation was conducted in reverse phase on a Phenomenex C18 column (5 µm, 250 × 4.6 mm), with the signal recorded using a photodiode array detector at 245 nm for ascorbic acid and at 215 nm for the other compounds. These detected molecules were identified by chromatographic comparisons with standards and quantified (mg/100 g fw and dw) by interpolating the peak areas in calibration curves (*r*^2^ ≥ 0.999) constructed with oxalic acid (*y* = 8 × 10^6^*x* + 331,789), ascorbic acid (*y* = 5 × 10^7^*x* + 449,262), and citric acid (*y* = 968,367*x* − 12,295) standards acquired from Sigma-Aldrich (St. Louis, MO, USA).

The sweetness index was calculated as the ratio of total soluble sugars to total organic acids.

#### 2.2.4. Lipophilic Constituents

The crude fat obtained by Soxhlet extraction was transesterified to obtain fatty acid methyl esters (FAME) for analysis in a YL 6500 gas chromatograph (Young In Chromass Co., Ltd., Anyang, Republic of Korea) equipped with a flame ionization detector, as described by Spréa et al. [30]. The injection was conducted in splitless mode, and chromatographic separation was achieved on a Zebron™ ZB-FAME column (0.20 µm, 30 m × 0.25 mm). Fatty acids were identified by chromatographic comparison of the retention times of the sample FAME peaks with those of the Supelco^®^ 37-component FAME mix (47885-U) from Sigma-Aldrich (St. Louis, MO, USA). In addition to the relative percentage of each fatty acid, the content (mg per 100 g fw and dw) was estimated based on the conversion factor (0.8) proposed by Greenfield and Southgate [31].

Tocopherols were analyzed using the Knauer HPLC coupled to a Jasco FP-2020 intelligent fluorescence detector, programmed for excitation at 290 nm and emission at 330 nm, as described by Pinela et al. [28]. The powdered berry sample was mixed with tocol (50 μg/mL) and extracted with methanol, *n*-hexane, and a saturated NaCl solution. After collecting the lipophilic fraction, the extraction was repeated twice with hexane. The extracts were dried under a nitrogen stream, redissolved in *n*-hexane, and filtered through a 0.22 μm filter disk. Normal-phase chromatographic separation was conducted on a YMC-Pack Polyamine II column (5 μm, 250 × 4.6 mm). Tocopherols were identified by chromatographic comparisons with authentic standards and quantified (mg per 100 g fw and dw) using the internal standard method.

### 2.3. Preparation of Fruit and Calyx Extracts

Powdered fruit and calyx samples (~1 g) were subjected to dynamic maceration twice using 80% ethanol (30 mL) for 1 h at room temperature. The resulting supernatants were filtered through Whatman no. 4 filter paper and concentrated under reduced pressure [32]. This traditional method is widely used to extract phenolic compounds from plant matrices due to the simplicity and efficiency of the hydroethanolic solvent. Furthermore, a decoction of the calyx was prepared following traditional folk medicine practices to further explore this herbal preparation [33]. For this, the calyx sample (1 g) was boiled in 200 mL of distilled water for 5 min, allowed to stand at room temperature for another 5 min, and then filtered through Whatman no. 4 filter paper. The aqueous mixtures were subsequently freeze-dried to obtain dry extracts for further analysis.

### 2.4. Analysis of Phenolic Compounds in Fruit and Calyx Extracts

The dried extracts were dissolved in ethanol/water (20:80, *v*/*v*) at 5 mg/mL, filtered through 0.22-μm filter disks, and subsequently analyzed for phenolic compounds using a Thermo Scientific Ultimate 3000 HPLC system [34]. A Waters Spherisorb^®^ S3 ODS-2 C18 column (3 µm, 4.6 mm × 150 mm) was used for compound separation, and detection was conducted using a diode array detector (280 and 370 nm) and a Thermo Scientific™ LTQ XL™ linear ion trap mass spectrometer with an electrospray ionization source (Table S2). Compound identification involved comparing their retention time and UV-Vis and mass spectra with those of commercial standards. In cases where standards were unavailable, chromatographic data were compared with those reported in the literature. Compounds were quantified (mg per g of extract) using calibration curves constructed with the standards listed in Table S3; thus, the results were expressed in mg equivalents of the most similar compound.

### 2.5. Evaluation of Biological Activities of Fruit and Calyx Extracts

#### 2.5.1. Antioxidant Activity

The antioxidant properties of the extracts were assessed through two cell-based in vitro methodologies described by Añibarro-Ortega et al. [35]. These assays evaluated the extracts’ ability to inhibit the formation of malondialdehyde (MDA) and other reactive substances and to prevent oxidative hemolysis.

*TBARS assay.* A suspension of porcine brain cells (1:2, *w*/*v*) prepared in an ice-cold Tris–HCl buffer (20 mM, pH 7.4) was incubated at 37 °C with extract (0.08–10 mg/mL for fruit and 0.04–5 mg/mL for calyx) or Trolox (3.125–100 µg/mL) in the presence of FeSO_4_ (10 µM) and ascorbic acid (0.1 mM). After 1 h, the reaction was halted by adding trichloroacetic acid (28% *w*/*v*). Thiobarbituric acid (TBA, 2% *w*/*v*) was then added, and the mixture was heated to 80 °C for 20 min to allow for the formation of MDA-TBA_2_ adducts monitored at 532 nm. The efficacy was reported as EC_50_ values (µg/mL), representing the extract concentration required to inhibit 50% of TBARS formation.

*OxHLIA assay*. A suspension of sheep red blood cells (2.8%, *v*/*v*) prepared in phosphate-buffered saline (PBS, pH 7.4) was mixed with either the extract (0.44–7 mg/mL for fruit and 12.5–400 µg/mL for calyx), Trolox (3.125–250 μg/mL), PBS as the negative control, or distilled water as a baseline control. Following a 10-min pre-incubation at 37 °C with shaking, 2,2′-azobis(2-amidinopropane) dihydrochloride (160 mM) was added, and the optical density was recorded at 690 nm over 400 min. IC_50_ values (µg/mL) were determined for time intervals (Δ*t*) of 60, 120, and 180 min. These values represent the extract concentration required to protect 50% of the red blood cell population from oxidative hemolysis during the specified time intervals.

#### 2.5.2. Antidiabetic Potential

The inhibitory effects of the extracts on yeast α-glucosidase activity and the formation of advanced glycation end products (AGEs), directly linked to their potential as antidiabetic agents, were evaluated using the protocols described by Millán-Laleona et al. [36]. Acarbose and aminoguanidine were the positive controls for α-glucosidase and AGE formation inhibition, respectively. The results were expressed as IC_50_ values (µg/mL).

#### 2.5.3. Anti-Obesity Potential

The lipase inhibition assay followed the procedure described by Millán-Laleona et al. [36], with orlistat as the positive control. The results were reported as IC_50_ values (µg/mL).

#### 2.5.4. Nitric Oxide Production Inhibition Capacity

The inhibitory activity of the extracts (0.625–400 µg/mL) was assessed by measuring their effect on nitric oxide (NO) production by lipopolysaccharide (LPS)-stimulated RAW 264.7 macrophages acquired from the European Collection of Authenticated Cell Cultures (ECACC). Nitrite (NO_2_^−^) levels in the culture medium, indicative of NO production, were quantified using a Griess reagent system kit and calibrated against a NO_2_^−^ standard curve (*y* = 0.0068*x* + 0.0951, *r*^2^ = 0.9864) [37]. Dexamethasone (7.65–980 µg/mL) was used as a positive control, and samples with and without LPS served as negative controls. The results were reported as EC_50_ values (µg/mL).

#### 2.5.5. Antiproliferative Activity

The antiproliferative activity of the extracts (6.24–400 µg/mL) was evaluated on human tumor cell lines acquired from the Leibniz Institute DSMZ, specifically AGS (gastric adenocarcinoma), Caco-2 (colorectal adenocarcinoma), MCF-7 (breast adenocarcinoma), and NCI-H460 (non-small-cell lung carcinoma). Additionally, a non-tumor porcine liver primary cell culture (PLP2) was included in the study. The sulforhodamine B assay was followed according to previously described protocols, with ellipticine (0.38–12.3 µg/mL) as the positive control [38,39]. The results were reported as GI_50_ values (μg/mL).

#### 2.5.6. Antimicrobial Activity

The extracts, reconstituted in 30% ethanol, were tested against the foodborne bacteria and fungi listed in Table S1. Minimum inhibitory concentrations (MIC) and minimum bactericidal (MBC) or fungicidal (MFC) concentrations (mg/mL) were determined following previously described serial dilution methods [40,41]. Sodium benzoate and potassium metabisulfite were the positive controls, while 30% ethanol was the negative control.

### 2.6. Statistical Analysis

Results from at least three independent experiments are presented as mean ± standard deviation (SD), except for antimicrobial activity results, for which MIC, MBC, or MFC are reported. The decimal place of the mean value’s uncertain digit was established by rounding the SD to one significant figure. For the OxHLIA, α-glucosidase activity inhibition, AGEs formation inhibition, and lipase inhibition assays, non-linear regression analysis was performed using GraphPad Prism^®^ 8 (GraphPad Software, San Diego, CA, USA) to fit dose-response curves and calculate IC_50_ values with 95% confidence intervals. Statistical differences between two dependent variables were assessed using a two-tailed paired Student’s *t*-test. For comparisons involving three or more dependent variables, one-way analysis of variance (ANOVA) was performed, employing Tukey’s HSD test for homoscedastic data (*p* > 0.05) and Tamhane’s T2 test for heteroscedastic data (*p* < 0.05). All statistical tests were performed at a 5% significance level using SPSS Statistics software (IBM SPSS Statistics for Windows, Version 22.0. IBM Corp., Armonk, NY, USA).

## 3. Results and Discussion

### 3.1. Nutrient Composition of the Goldenberry Fruit

#### 3.1.1. Proximate Composition and Energy

The *P. peruviana* fruit is commonly consumed fresh and used in the food industry to produce beverages, compotes, jams, and pastry products such as pies and cakes. It is also available in the dried form and as freeze-dried powder. Therefore, it is important to know the nutritional value of the fresh fruit and its freeze-dried powder. Table 1 presents the nutritional composition of the *P. peruviana* fruit. This berry contained 79 g/100 g of moisture, which is consistent with previous studies that reported moisture values ranging from 77 to 85 g/100 g [42,43,44,45]. In turn, the freeze-dried powder contained 5.4 g/100 g of moisture.

Carbohydrates were the most abundant macronutrients quantified in the fruit, followed by total dietary fiber, with ash and protein present in smaller quantities (Table 1). Petkova et al. described a total carbohydrate range from 10.23 to 14.13 g/100 g in Bulgarian samples, which aligns with the value observed in our sample, as well as a similar content (11.34 g/100 g) in fruits imported from Colombia [45]. In comparison, Pereda reported carbohydrate levels between 14.22 and 19.66 g/100 g in fruits from northern Argentina [46], which are slightly higher than the 11.90 g/100 g measured in this study.

The dietary fiber content reached 5.16 g/100 g, comparable to the 4.9 g/100 g reported by Ramadan [47]. In contrast, Pereda reported slightly lower crude fiber values (4.12–4.25 g/100 g) [46], a slight difference that may be attributed to methodological limitations. This method quantifies only the fiber insoluble in acid or alkaline solutions, thus not accounting for the total dietary fiber. According to current regulations [48], the nutritional claims “source of fiber” and “high in fiber” can be attributed to the *P. peruviana* fruit and its freeze-dried power, respectively. Therefore, consuming *P. peruviana* fruit in both fresh and dried forms can help achieve the recommended daily intake (RDI) of at least 25 g of dietary fiber recommended for adults by the European Food Safety Authority (EFSA). This recommendation is supported by evidence highlighting the benefits of dietary fiber for bowel function and the prevention of non-communicable diseases, including type 2 diabetes, colorectal cancer, cardiovascular diseases, and conditions such as overweight and obesity.

The *P. peruviana* fruit contained 1.75 g/100 g of ash (Table 1), slightly exceeding the previously reported range of 0.77–1.00 g/100 g in some studies [43,44,46]. In contrast, significantly higher ash values (~5.32 g/100 g) were described by Petkova et al. for three goldenberry genotypes [45]. The same authors also noted that the ash contents of the fruit pulp varied significantly, ranging from 2.42 to 4.23 g/100 g.

Regarding protein content, the fruit contained 1.37 g/100 g, a value within the previously reported range of 0.05–2.54 g/100 g [43,44,45,46]. Petkova et al. described a higher protein content (2.54 g/100 g) in a *P. peruviana* variety imported from Colombia compared to two genotypes produced in Bulgaria (~1.97 g/100 g) [45]. The protein content of *P. peruviana* fruit was comparable to that previously described for *fruta-do-lobo* (*Solanum lycocarpum* St. Hill) pulp, which also contains 1.37 g/100 g [49]. However, it exceeded the protein content of tomatoes (*S. lycopersicum* L.), which typically ranges from 0.40 to 0.75 g/100 g [49,50] but was lower than the 2.05 g/100 g described for *juá-açu* (*S. oocarpum* Sendtn.) pulp [49]. This comparison highlights the nutritional value of *P. peruviana* fruit, suggesting that its protein content is slightly higher than that of widely consumed tomatoes. These findings reinforce the potential of this berry for inclusion in modern diets and for the development of powder formulations or other dietary supplements aimed at enhancing protein intake.

A crude fat content of 0.492 g/100 g was found in *P. peruviana* fruit (Table 1), closely matching the 0.5 g/100 g reported by Pereda [46]. This value is higher than the 0.15–0.20 g/100 g documented by Ramadan [47], within the range of 0.25–1.01 g/100 g reported by Petkova et al., but lower than the 2.0 g/100 g and 3.16 g/100 g reported by Ramadan and Mörsel [51] and Rodrigues et al. [43], respectively. Ramadan and Mörsel also described the seeds, which make up approximately 17% of the edible fruit, as containing approximately 90% (1.8 g/100 g) of the total crude fat of the whole fresh berry, while the pulp and peel contribute only about 10% [44]. With a fat content below 0.5 g/100 g, *P. peruviana* fruit can be classified as a fat-free food [48].

A 100 g serving of fresh *P. peruviana* fruit provided a low energy value of 67.8 kcal (Table 1), with carbohydrates being the macronutrient that contributed the most to this value. This energy content is slightly higher than the 53 kcal/100 g reported in the USDA food composition database [52]. In comparison, the energy value of the freeze-dried fruit powder reached 307 kcal/100 g.

#### 3.1.2. Mineral Elements

The intake of mineral elements is vital for metabolic processes and the proper functioning of the human body. Ensuring that daily diets include food products that provide appropriate amounts of macrominerals and trace elements, among other nutrients, is essential for maintaining health. with their consumption promoted as a preventive measure against diseases and functional impairments.

As shown in Table 1, K was the most abundant mineral in *P. peruviana* fruit, followed by Mg, P, and Ca, and lower amounts of Na and trace elements. Musinguzi et al. [53] and Leterme et al. [54] similarly identify K (210 mg/100 g), Mg (7 mg/100 g), P (34 mg/100 g), and Ca (28 mg/100 g) as the predominant minerals in *P. peruviana* fruit. Petkova et al. also highlighted Bulgarian goldenberry genotypes as rich sources of K, with levels ranging from 388 to 496 mg/100 g [45], comparable in whole fruit and pulp but higher than those observed in the present study. According to EFSA, a 100-g portion of this orange-yellow fruit provides 7% of the RDI of K for adult female and male individuals, while the freeze-dried powder contributes 35% [55]. Potassium-rich foods are particularly important as this mineral is critical in regulating fluid balance, nerve signals, and muscle contractions. Furthermore, K helps maintain healthy blood pressure by counteracting the effects of Na, thereby reducing the risk of hypertension and heart disease [55]. Adequate K intake also supports bone health and reduces the likelihood of kidney stones, making it important for overall cardiovascular and metabolic well-being [56].

A 100 g portion of *P. peruviana* fruit also provides 10% of the RDI of Mn, 8% of P, 6% of Cu, 5–6% of Mg, 4% of Fe, and 2% of Zn for adult male and female individuals, and the freeze-dried powder 49% of Mn, 39% of P, 29–30% of Cu, 27% of Mg, 20–21% of Fe, and 11% of Zn calculated based on the recommendations of the EFSA [55]. Additionally, the consumption of freeze-dried powder from this citrus-flavored fruit as a food ingredient, dietary supplement, or nutraceutical could be an effective strategy to enhance the intake of these essential elements, for example, when dissolved in citrus-flavored beverages.

#### 3.1.3. Soluble Sugars and Organic Acids

The sugar and organic acid composition of fruits are critical determinants of their sensory attributes, nutritional value, and overall quality. Simple carbohydrates such as glucose, fructose, and sucrose primarily contribute to the sweetness and energy content. In contrast, organic acids such as citric and ascorbic acids play a vital role in flavor balance, preservation, and provision of health benefits. These compounds not only enhance the fruit’s taste but also influence its shelf life and nutritional profile, thereby significantly impacting consumer preference and appeal [57].

As shown in Table 2, the total soluble sugar concentration in *P. peruviana* fruit reached 9.9 g/100 g, with fructose being the most abundant sugar, followed by glucose and sucrose. This value is higher than the 3.27–5.88 g/100 g reported by Petkova et al. for two *P. peruviana* fruit genotypes from Bulgaria and an imported Colombian fruit sample or the 8.4 g/100 g listed in the USDA food composition database [52]. Petkova et al. also found similar levels of glucose and fructose between genotypes, while sucrose content varied more widely, probably in response to environmental conditions [45].

Organic acids were detected in a total concentration of 1.46 g/100 g (Table 2), with citric acid as the predominant compound (accounting for 97% of the total content), followed by ascorbic acid (32.0 mg/100 g) and oxalic acid (20.5 mg/100 g). A previous study reported higher levels of citric acid, ranging from 1.60 to 2.30 g/100 g, varying according to the altitude at which the plant was cultivated [58]. Ascorbic acid levels reported in earlier studies ranged from 14.5 mg/100 g in wild-collected fruits to 32.21 mg/100 g in cultivated fruits of *P. peruviana* from the Argentinean northern Andean region [46]. Notably, a level of 32.2 mg/100 g, matching the value observed in this study, was quantified in *P. peruviana* ecotypes grown in Colombia [58], which also contained malic and tartaric acids. Additionally, ascorbic acid concentrations between 30 and 40 mg/100 g were detected in *P. peruviana* fruit juice [59].

The calculated sweetness index (sugars/acids ratio) for *P. peruviana* fruit was 7.1, which falls within the range of 3.48 to 9.27 reported for goldenberries of Bulgarian and Colombian origin [45]. This confirms the fruit’s characteristic sweet-sour taste profile. According to established criteria, a sweetness index below 5 would classify the fruit as having a predominantly sour-sweet taste [45].

#### 3.1.4. Fatty Acids and Tocopherols

Despite the low crude fat content found in *P. peruviana* fruit, the characterization of its fatty acid profile offers valuable insights into its nutritional and health benefits, particularly due to the presence of unsaturated fatty acids, as well as its potential functional applications in food product development. As shown in Table 3, the crude fat content of *P. peruviana* fruit was primarily composed of polyunsaturated fatty acids (PUFA), with linoleic acid (C18:2*n*-6C) being the most abundant, accounting for 70% of the total fatty acids, or 43.3 mg/100 g of the fresh fruit. The monounsaturated fatty acid (MUFA) oleic acid (C18:1*n*-9C) ranked second (11.0%), followed by the saturated fatty acids (SFA) palmitic acid (C16:0; 10.9%) and stearic acid (C18:0; 4.0%). This trend aligns with reports in the literature, which indicate a predominance of linoleic acid (70.5–72.4%), followed by oleic (10.0–13.0%), palmitic (8.62–9.38%), and stearic (2.57–2.67%) acids in *P. peruviana* fruit samples from Brazil and Colombia [51]. According to Ramadan and Mörsel, approximately 90% of the lipid fraction in this fruit is contained in its yellowish seeds [51]. Linoleic acid, an essential *n*-6 PUFA, is critical in reducing the risk of cardiovascular disorders, including coronary heart disease, atherosclerosis, and high blood pressure [60]. As a result, foods rich in linoleic acid should be part of a healthy diet, but balance and moderation are important. Ensuring a good ratio of *n*-6 to *n*-3 fatty acids is critical for optimal nutrition.

The tocopherol composition of *P. peruviana* fruit is closely linked to its nutritional and functional properties, as tocopherols are vitamin E components and contribute to the fruit’s antioxidant capacity. As shown in Table 4, the total tocopherol concentration in *P. peruviana* fruit reached 2.34 mg/100 g, with γ-tocopherol as the most abundant isoform, followed by α- and β-tocopherols.

Previous studies have focused on analyzing tocopherol content in *P. peruviana* oils rather than in the whole fruit. Ramadan and Mörsel identified β-and γ-tocopherols as the predominant isoforms in whole berry oil [51]. Similarly, Popova et al. reported notable differences in the tocopherol profile of seed oils, observing that β- and γ-tocopherols increase approximately three and eight times, respectively, as the fruit matures [61]. In contrast, a study on *P. peruviana* fruits grown in Chile identified α-tocopherol as the dominant isomer in pressed and homogenized pulp samples [62]. These findings highlight how the tocopherol profile varies depending on the fruit part analyzed and the stage of maturity.

The total tocopherol content quantified in *P. peruviana* fruit was higher than the levels (1.02–1.44 mg/100 g) previously reported for fresh table tomato farmer varieties [50]. This elevated tocopherol concentration, combined with the presence of PUFA, highlights goldenberries as a valuable dietary source of essential micronutrients. As powerful antioxidants, tocopherols protect cells from oxidative damage and support immune system function [63]. PUFA, such as linoleic and α-linolenic acids, play a crucial role in cardiovascular health by lowering LDL cholesterol levels, reducing inflammation, and supporting brain function [64]. The presence of these constituents in goldenberry suggests that its inclusion in the diet may help reduce the risk of some chronic diseases, including cardiovascular conditions, and support overall well-being.

### 3.2. Phenolic and Steroidal Constituents of Goldenberry Fruit and Calyx Extracts

The phenolic and steroidal profiles of *P. peruviana* fruit and calyx extracts were characterized by HPLC-DAD-ESI/MS*^n^*, with the resulting chromatograms shown in Figure S1. The chromatographic retention times, maximum absorption wavelengths (λ_max_), deprotonated molecules ([M − H]^−^/[M]^−^), and main fragment ions in tandem MS^2^ used for tentative identification of compounds, as well as the quantitative results, are presented in Table 5. Seven withanolides were tentatively identified in the fruit extract, while hydroethanolic and decocted calyx extracts contained three phenolic acids and four flavonoids.

Compounds **1** and **2** showed a deprotonated molecule at *m*/*z* 619, with the main fragment ion at *m*/*z* 573, and were tentatively identified as daturametelin N, a withanolide previously reported in other Solanaceae species, particularly in *Datura metel* L. flower, leaf, stem, root, seed, and peel [65]. Compound **3** was tentatively identified as 3-methoxy-7-hydroxy-6-deoxyphysalin D, a compound already reported in the calyx of *Physalis alkekengi* L. [66], due to its deprotonated molecule at *m*/*z* 575, main fragment ion at *m*/*z* 543, and minor fragments at *m*/*z* 529 and 253. Compound **4** showed a deprotonated molecule at *m*/*z* 557, with a main fragment ion at *m*/*z* 471 and less abundant ions at *m*/*z* 495, 323, and 121, leading to its tentative identification as 3-methoxyphysalin A [66]. Compound **5** was tentatively identified as 4,7-didehydroneophysalin B base on a deprotonated molecule at *m*/*z* 509 and fragment ions at *m*/*z* 463 and 491 [66]. Compound **6** had a molecular ion at *m*/*z* 543, with main fragment ions at 497, 499, and 453, leading to its tentative identification as physalin E [66]. Compound **7**, with a deprotonated molecule at *m*/*z* 539, a main fragment ion at *m*/*z* 511, and less abundant fragments at *m*/*z* 345 and 495, was tentatively identified as 3-methoxy-6,7,9,10-tetradehydrophysalin B [66]. While the identified physalins have been previously reported in *P. peruviana* fruit extracts [69], this was the first time a daturafoliside was identified in this species.

Compounds **8**, **9**, and **10** were tentatively identified as caffeoylquinic acids (Table 5). All exhibited a deprotonated molecule at *m*/*z* 353, with a main fragment ion at *m*/*z* 191. Compound **8** also showed less abundant fragment ions at *m*/*z* 179 and 173, compound **9** at *m*/*z* 179, 173, and 135, and compound **10** at *m*/*z* 179 and 173. Based on the retention times and fragment data, the compounds were identified as 1-*O*-, *trans*-5-*O*-, and *cis*-5-*O*-caffeoylquinic acids, respectively [67,68]. Compounds **11**, **12**, **13**, and **14** exhibited deprotonated molecules at *m*/*z* 609, with a main fragment ion at *m*/*z* 301. Compound **13** was tentatively identified as rutin (quercetin-3-*O*-rutinoside) based on its chromatographic comparison with the standard. In contrast, compounds **11**, **12**, and **14** were assigned the generic designation of quercetin-deoxyhexosyl-hexoside, as the sugar moiety could not be confirmed in the reference used for identification. The presence of polyphenols such as caffeoylquinic acid and quercetin-3-*O*-glucoside in *P. peruviana* calyx was previously documented [70,71].

In the calyx extracts, the sum of all phenolic compounds reached 42.0 ± 0.2 mg/g hydroethanolic extract and 28.2 ± 0.1 mg/g decocted extract. Flavonoids were the most abundant compound class, with concentrations of 31.2 ± 0.1 mg/g in the hydroethanolic extract and 19.7 ± 0.4 mg/g in the decocted extract. Phenolic acids were found in lower concentrations, with 10.84 ± 0.05 mg/g in the hydroethanolic extract and 8.5 ± 0.1 mg/g in the decocted extract. Therefore, the hydroethanolic extraction was more effective than the aqueous decoction in recovering these secondary metabolites from the papery calyx.

In the fruit, although the identified compounds could not be quantified due to the lack of available standards, it is worth noting that, based on the peak areas (Figure S1a), the major compound seemed to be 3-methoxyphysalin A.

### 3.3. Bioactive Properties of Goldenberry Fruit and Calyx Extracts

#### 3.3.1. Antioxidant Activity

The antioxidant activity results for the *P. peruviana* fruit and calyx extracts are presented in Table 6. The OxHLIA assay demonstrated that the *P. peruviana* fruit hydroethanolic extract exhibits antioxidant activity by protecting erythrocytes from oxidative hemolysis initiated by the peroxyl radicals. These radicals are generated in the system through the thermal decomposition of 2,2′-azobis(2-amidinopropane) hydrochloride in the presence of oxygen [72]. Peroxyl radicals induce the oxidation of PUFA in erythrocyte membranes, triggering a chain reaction known as lipid peroxidation [72]. This process leads to rapid membrane damage and losses of structural integrity. When antioxidants are present, they can scavenge peroxyl radicals and convert them to non-reactive species, thereby inhibiting hemolysis. A 594 µg/mL concentration of fruit extract protected 50% of the erythrocyte population from the free radical-induced oxidative damage for 60 min, while 880 µg/mL and 1166 µg/mL provided similar protection for 120- and 180-min intervals, respectively. Similarly, the TBARS assay revealed that 3493 µg/mL of the same extract inhibited 50% of the lipid peroxidation, as determined by measuring the formation of MDA-TBA_2_ adducts. These findings suggest that *P. peruviana* fruit exerts in vitro antioxidant effects against lipidic peroxidation. These effects may be attributed to bioactive compounds such as phenolics, withanolides, organic acids (e.g., ascorbic acid), and tocopherols. However, the positive control demonstrated significantly higher activity than the extract, which may be explained by the fact that the fruit contains a large amount of carbohydrates that dilute the concentration and efficacy of the antioxidants.

Significant differences were observed between the samples and the positive control (Table 6) regarding the calyx extracts. The hydroethanolic extract was more effective in inhibiting oxidative hemolysis, with IC_50_ values ranging from 42 to 145 ± 2 µg/mL for 60 to 180-min intervals, compared to the decocted extract, which yielded IC_50_ values between 60 and 170 µg/mL over the same time intervals. These results indicate that both extraction methods provide extracts with good antioxidant activity against free radical-induced hemolysis, even though they are relatively less active than the positive control, trolox. In contrast, for the TBARS formation inhibition, the decocted extract showed significantly higher antioxidant activity than the hydroethanolic extract, which was 14.3% less bioactive. Nonetheless, both extracts demonstrated considerable antioxidant potential, although neither was as effective as trolox, which achieved an EC_50_ of 5.4 µg/mL.

These findings suggest that extracts derived from *P. peruviana* calyces exhibit significant antioxidant activity, possibly due to their rich composition of phenolic compounds such as phenolic acids and flavonoids. Therefore, *P. peruviana* calyx emerges as a promising source of bioactive functional ingredients with potential applications in the food, nutraceutical, and related industries to mitigate oxidative reactions, particularly in PUFA-rich foods prone to rancidity, thereby preserving quality attributes and extending shelf life. To the best of the authors’ knowledge, this study is the first to employ cell-based OxHLIA and TBARS assays to evaluate the antioxidant activity of *P. peruviana* extracts.

#### 3.3.2. Antidiabetic Potential

Foods containing bioactive compounds that inhibit α-glucosidase activity and AGE formation can be highly beneficial for human health. By inhibiting α-glucosidase, such foods slow down the digestion and absorption of carbohydrates, which helps prevent rapid spikes in blood glucose levels. This is particularly useful for managing diabetes and improving glycemic control, reducing the risk of diabetes-related complications [73]. On the other hand, AGEs are harmful compounds formed when proteins or fats combine with sugars, leading to oxidative stress and inflammation. High levels of AGEs are associated with chronic diseases such as diabetes, cardiovascular disease, and neurodegenerative disorders. Foods that inhibit AGE formation help reduce these risks by mitigating oxidative damage and inflammatory responses [73].

**Figure 1:**
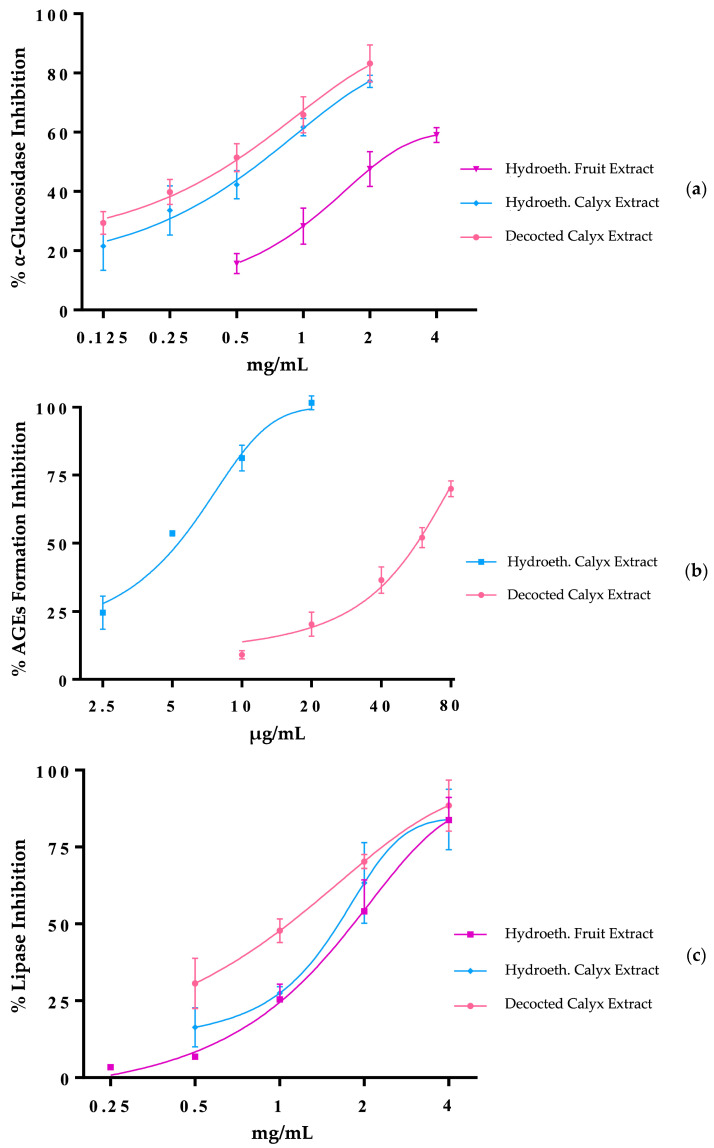
Inhibitory capacity of *P. peruviana* fruit and calyx extracts on (**a**) α-glucosidase activity, (**b**) AGEs formation, and (**c**) lipase activity.

Table 6 and Figure 1a show that the *P. peruviana* fruit extract inhibited α-glucosidase activity with an IC_50_ value of 2548 µg/mL. This value is nearly seven times higher than that of acarbose, a well-established reference inhibitor of α-glucosidase, which yielded an IC_50_ value of 380 µg/mL. Although the comparison is between a natural crude extract and a synthesized drug, the result underscores the potential functional effects of goldenberry. The hydroethanolic and decocted calyx extracts showed IC_50_ values of 780 and 784 µg/mL, respectively. These values were three times lower than that of the fruit and only two times higher than that of the synthetic control, demonstrating the higher efficacy of the underutilized calyx.

While the fruit extract did not inhibit AGE formation, the calyx extracts showed significant activity. The decocted calyx extract had an IC_50_ value of 70 µg/mL, comparable to that of aminoguanidine, while the hydroethanolic extract exhibited an IC_50_ value of 6 µg/mL, making it more than ten times as potent as the positive control (Table 6, Figure 1b). This higher activity may be attributed to the higher levels of phenolic acids and flavonoids in the hydroethanolic extract (Table 5). These results highlight the strong potential of calyx extracts in inhibiting glycation—a complex, non-enzymatic process initiated by interaction between the carbonyl group of a reducing sugar and a free amino group.

Previous studies, such as those by Pinto et al., have noted *P. peruviana* for its moderate α-glucosidase inhibitory activity and high phenolic content, which contribute to its antioxidant potential. These properties support its role in managing hyperglycemia through enzyme inhibition and oxidative stress reduction [74]. Tshibangu et al. demonstrated that *P. peruviana* fruit methanolic extracts exhibit high antidiabetic activity in both in vitro and in vivo experiments. Their research showed significant reductions in hyperglycemia in glucose-induced diabetic mice, supported by molecular docking studies that revealed strong interactions with α-glucosidase and insulin receptor kinase domains [75]. Similar antidiabetic effects were observed by Ezzat et al., who reported improved insulin sensitivity in streptozotocin-induced diabetic rats treated with *P. peruviana* fruit hydroethanolic extract and its ethyl acetate fraction [22].

Recent studies have highlighted the antidiabetic potential of *P. peruviana* calyces. For instance, a butanol fraction derived from calyces improved glucose regulation, insulin resistance, and oxidative stress markers in streptozotocin-induced diabetic mice [21]. Persistent hyperglycemia exacerbates oxidative stress and accelerates AGE formation, leading to complications such as nephropathy and retinopathy. Aljadani et al. demonstrated that goldenberry juice reduced oxidative stress markers and AGE levels in diabetic rats, providing nephroprotective effects by mitigating inflammation and enhancing antioxidant enzyme activity [76]. These findings align with the observed activity of calyx extracts, suggesting that their functional benefits extend beyond enzymatic inhibition [76]. Additionally, Gironés-Vilaplana et al. analyzed the polyphenolic composition and bioactivities of *P. peruviana* fruit and calyx extracts. The fruit extract showed significant α-glucosidase inhibitory activity, attributed to its rich phenolic content, including flavonoids and phenolic acids, which are known to delay glucose absorption by inhibiting carbohydrate-digesting enzymes [77]. The study also noted the moderate antioxidant capacity of calyx extracts, further supporting their antidiabetic potential.

The significant ability of the calyx extracts to inhibit both α-glucosidase activity and AGE formation positions them as a promising natural source of potential anti-diabetic agents. Their dual action suggests they could serve as valuable functional ingredients for developing functional foods, food supplements, and related products, particularly for managing diabetes and mitigating its complications. Further research and development could establish their efficacy and broaden their applications in dietary interventions to enhance metabolic health.

Evidence also highlights the antidiabetic potential of *P. peruviana* leaves. Kasali et al. found that hydromethanolic and dichloromethane-methanol fractions of leaf extracts exhibit significant α-glucosidase inhibitory activity, with IC_50_ values comparable to those of standard antidiabetic drugs [78]. The antioxidant properties of these fractions, demonstrated through radical scavenging assays, underscore their capacity to mitigate oxidative stress. These findings suggested that the leaves represent another promising part of the plant for diabetes management, warranting further investigation [78].

#### 3.3.3. Anti-Obesity Potential

Inhibiting lipase activity in foods is an effective strategy for managing body weight and improving metabolic health. Lipase inhibitors reduce dietary fats’ digestion and absorption, lowering calorie intake. These effects help mitigate the risk of obesity and cardiovascular diseases [79]. As shown in Table 6 and Figure 1c, the hydroethanolic extracts of *P. peruviana* fruit and calyx demonstrated lipase inhibitory activity, with IC_50_ values ranging from 2055 to 2200 µg/mL. Notably, the decocted calyx extract exhibited a stronger lipase inhibition capacity (IC_50_ of 1288 µg/mL) than the hydroethanolic extracts. Although orlistat, a synthetic lipase inhibitor, displayed a much stronger inhibitory effect (IC_50_ value of 40 µg/mL), the calyx extracts still showed considerable potential as a natural alternative for managing lipid metabolism. The differences in inhibitory capacity among the extracts may reflect the contribution of bioactive compounds other than phenolics, even though hydroethanolic extraction yielded higher levels of phenolic compounds.

A study evaluating the lipase inhibitory activity of *P. peruviana* further reinforced the potential of its fruit and calyx extracts. The hydroethanolic calyx extract displayed notable inhibitory capacity, highlighting its potential as a functional ingredient for managing lipid metabolism and addressing obesity-related issues [77]. The findings underscore the significance of *P. peruviana* bioactive compounds in modulating enzyme activity and suggest its potential for developing natural alternatives in health and nutrition.

In addition to lipase inhibition, *P. peruviana* extracts also demonstrated the ability to inhibit lipid peroxidation and α-glucosidase activity and suppress the formation of AGEs. This multifaceted functionality is particularly significant, as it targets multiple metabolic pathways. By alleviating oxidative stress, mitigating hyperglycemia, and reducing fat absorption, *P. peruviana* calyx extracts present a holistic approach to managing metabolic disorders such as diabetes and obesity. These findings position goldenberry calyces as a promising natural product for enhancing metabolic health and supporting weight management. Its potential applications in functional foods and dietary interventions make it a promising candidate for promoting consumer health and wellness.

#### 3.3.4. Anti-Inflammatory Potential

Chronic inflammation is associated with a range of serious diseases, including cardiovascular disease, diabetes, and cancer. One emerging strategy to address this inflammation and its related risk factors is incorporating antioxidant- and anti-inflammatory-rich foods into the diet. These functional foods play a crucial role in reducing chronic inflammation, alleviating symptoms of inflammatory disorders, supporting immune function, and improving digestive health [80]. Regularly consuming such foods can lower the risk of chronic diseases and promote overall well-being [81]. In this context, the hydroethanolic extract of *P. peruviana* fruit showed no inhibition of NO production by LPS-stimulated RAW 264.7 cells at concentrations up to 400 µg/mL. In contrast, the calyx extracts exhibited potent NO production inhibition capacity, surpassing that of the positive control, dexamethasone. The hydroethanolic extract at 40 µg/mL and the decocted extract at 32 µg/mL demonstrated NO production inhibition effects equivalent to 16 µg/mL of dexamethasone, a well-known steroidal anti-inflammatory agent.

Other studies have also highlighted the significant anti-inflammatory properties of *P. peruviana* calyx extract, such as in cases of renal dysfunction caused by Cd intoxication [19] and in colitis included by TNBS (2,4,6-trinitrobenzene sulfonic acid) in rats [82]. Moreover, withanolides isolated from *P. peruviana* calyces have strongly inhibited NO production in LPS-induced inflammatory models. Compounds such as peruvianolide C and D exhibited stronger inhibitory effects than standard anti-inflammatory agents, suggesting their potential for treating inflammation-related neurodegenerative disorders and chronic conditions [83].

The antioxidant and anti-inflammatory properties of *P. peruviana* calyx position it as a valuable natural product for managing inflammatory disorders and reducing the risk of chronic diseases. Its bioactive phytoconstituents not only neutralize species but also suppress key inflammatory mediators. This dual action offers an advantage for health interventions targeting oxidative stress and inflammation, enhancing its potential for use in the formulations of functional foods and dietary supplements.

#### 3.3.5. Antiproliferative Activity

As shown in Table 6, the *P. peruviana* fruit extract exhibited capacity to inhibit the proliferation of two human tumor cell lines, namely Caco-2 (colorectal adenocarcinoma) with a GI_50_ of 48 µg/mL and NCI-H460 (non-small-cell lung carcinoma) with a GI_50_ of 127 µg/mL. Although these values are significantly higher than those obtained with the positive control ellipticine (0.8 and 1.01 µg/mL, respectively), they still represent noteworthy antiproliferative activity. Comparatively, a previous study reported a GI_50_ value of 3.73 mg/mL for the ethanolic extract of the fruit against the Caco-2 cell line [84], nearly 80 times higher than the value obtained in this study. Notably, the *P. peruviana* fruit extract exhibited no activity against the PLP2 non-tumor cell culture.

For the calyx extracts, although neither surpassed the antiproliferative capacity of the positive control, both demonstrated significant activity against the tested cell lines. The decocted extract showed greater activity than the hydroethanolic extract for the AGS, Caco-2, MCF-7, and PLP2 cell lines. However, no significant differences were observed between the extracts in their effect on NCI-H460 tumor cells.

Although previous research has suggested that *P. peruviana* calyx exhibits hepatoprotective and nephroprotective properties [19,20], further investigation is necessary to elucidate the mechanisms of action and determine the toxicity thresholds for non-tumor cells, ensuring the safe and effective application of these extracts in therapeutic contexts.

#### 3.3.6. Antimicrobial Activity

The antimicrobial activity of *P. peruviana* fruit and calyx extracts was evaluated against foodborne bacterial and fungal strains. The results, summarized in Table 7, were compared to the antimicrobial effects of common food preservatives: sodium benzoate (a widely used food preservative also known as E211) and potassium metabisulfite (a food additive with antimicrobial properties known as E224).

The *P. peruviana* fruit hydroethanolic extract demonstrated particularly strong effectiveness against *S. aureus* and *B. cereus*, exhibiting superior antibacterial activity compared to sodium benzoate and potassium metabisulfite, respectively (Table 7). This extract also displayed significant activity against *Aspergillus* spp., *Penicillium* spp., and *Trichoderma* sp. strains, with MIC values (≤0.79 mg/mL) generally lower than those of both positive controls and MBC values (≤1.57 mg/mL) lower than those of sodium benzoate, highlighting its dual antibacterial and antifungal potential.

As shown in Table 7, the *P. peruviana* calyx extracts exhibited distinct antimicrobial profiles. The hydroethanolic extract demonstrated notable effectiveness against the bacteria *S. aureus* and *E. coli* and the fungi *P. verrucosum* var. *cyclopium* and *T. viride*. In contrast, the decocted extract showed superior activity against *L. monocytogenes*, *S. enterica* subsp. *enterica* ser. Typhimurium, and *A. fumigatus*. Compared to the positive controls, both extracts displayed greater activity against *B. cereus* and *A. versicolor* (MIC of 0.38 mg/mL and MBC of 0.75 mg/mL) than potassium metabisulfite. Furthermore, the hydroethanolic extract demonstrated greater antimicrobial activity against *S. aureus*, *E. coli*, *E. cloacae*, and five of the six tested fungal strains than sodium benzoate. Similarly, the decocted extract displayed notable efficacy against *S. aureus*, *S. enterica* subsp. *enterica* ser. Typhimurium, and five fungal species.

The calyx extracts demonstrated greater antimicrobial activity than the fruit extract, although both exhibited comparable antifungal efficacy. These findings highlight their potential as natural preservative agents, particularly in light of the increasing demand for clean-label ingredients in food formulations. Previous studies already described notable antibacterial effects of *P. peruviana* fruit ethanolic extracts against *B. cereus*, *E. coli*, *Pseudomonas typhimureum*, and *P. syringae*, as well as antifungal activity against *A. niger* and *Candida albicans* [84,85]. Future research would be important to identify the bioactive compounds responsible for these effects and to elucidate their underlying mechanisms of action.

## 4. Conclusions

This study examined the chemical composition and bioactive properties of *P. peruviana* fruit and calyx cultivated in Portugal, revealing their potential as nutrient-rich and/or health-promoting ingredients. The fruit was notably rich in carbohydrates (with 83.2% consisting of soluble sugars such as fructose and glucose) and total dietary fiber. Although low in fat, the *n*-6 PUFA linoleic acid accounted for 70% of the fatty acids. Additionally, the fruit provided significant amounts of ascorbic acid, tocopherols, and essential minerals, including K, Mn, P, and Mg. The freeze-dried fruit powder emerged as a concentrated source of micronutrients, making it a promising functional ingredient for fortifying food products and citrus-flavored beverages. Phytochemical analysis of the fruit extract identified five physalin derivatives and one withanolide, which exhibited significant antiproliferative capacity against human colorectal adenocarcinoma (Caco-2) and non-small-cell lung carcinoma (NCI-H460) cells. In turn, the calyx extracts contained three phenolic acids and four flavonoids, demonstrating high antioxidant activity through cell-based assays, inhibition of AGE formation, suppression of NO production, and antiproliferative activity against the tested tumor cell lines.

Overall, goldenberry was characterized as a nutrient-dense food, with its freeze-dried powder standing out as a concentrated source of minerals, vitamins, and other bioactive compounds suitable for use as a functional ingredient in various products. Future research should elucidate the mechanisms of action underlying these bioactive effects of the extracts and determine the toxicity threshold to ensure safe and effective applications. These findings underscore the potential of *P. peruviana* as a health-promoting fruit and highlight innovative opportunities for its calyx in the food, pharmaceutical, and nutraceutical industries, particularly in addressing oxidative stress-mediated and metabolic diseases.

## Figures and Tables

**Table 1:** Proximate composition, energy, and mineral composition of goldenberry fruit and freeze-dried powder.

Constituents	Content (per 100 g)	
	Fresh Fruit	Freeze-Dried Powder
Moisture (g)	79 ± 1	5.4 ± 0.2
Proteins (g)	1.37 ± 0.05	6.7 ± 1.3
Ash (g)	1.75 ± 0.06	8.4 ± 0.3
Fat (g)	0.492 ± 0.007	2.38 ± 0.03
Dietary fiber	5.2 ± 0.1	25.0 ± 0.5
Carbohydrates (g)	11.9 ± 0.1	52.2 ± 0.6
Energy (kcal)	67.8 ± 0.2	307 ± 1
K (mg)	253 ± 6	1222 ± 39
Na (mg)	1.41 ± 0.02	6.8 ± 0.1
Ca (mg)	3.0 ± 0.2	14.5 ± 0.7
Mg (mg)	19.9 ± 0.7	96 ± 3
Mn (mg)	0.31 ± 0.02	1.48 ± 0.08
Zn (mg)	0.221 ± 0.004	1.07 ± 0.02
Fe (mg)	0.53 ± 0.03	2.6 ± 0.2
Cu (mg)	0.094 ± 0.004	0.46 ± 0.01
P (mg)	45 ± 1	216 ± 6

The results are reported as mean ± standard deviation.

**Table 2:** Free sugar and organic acid composition of goldenberry fruit and freeze-dried powder.

Constituents	Content (per 100 g)	
	Fresh Fruit	Freeze-Dried Powder
Fructose (g)	4.1 ± 0.2	19.7 ± 0.9
Glucose (g)	3.5 ± 0.2	17 ± 1
Sucrose (g)	2.4 ± 0.1	11.4 ± 0.6
Σ Soluble sugar (g)	9.9 ± 0.5	48 ± 2
Oxalic acid (mg)	20.5 ± 0.5	99 ± 2
Ascorbic acid (mg)	32.0 ± 0.3	155 ± 1
Citric acid (g)	1.41 ± 0.01	6.82 ± 0.07
Σ Organic acids (g)	1.46 ± 0.02	7.07 ± 0.07

The results are reported as mean ± standard deviation.

**Table 3:** Fatty acid composition of goldenberry fruit and freeze-dried powder.

Constituents	Relative Percentage (%)	Content (mg/100 g) *	
		Fresh Fruit	Freeze-Dried Powder
C10:0	0.57 ± 0.02	2.26 ± 0.06	10.9 ± 0.3
C12:0	0.166 ± 0.004	0.65 ± 0.02	3.15 ± 0.08
C14:0	0.201 ± 0.004	0.79 ± 0.02	3.82 ± 0.08
C15:0	0.117 ± 0.004	0.46 ± 0.01	2.22 ± 0.07
C16:0	10.9 ± 0.2	42.8 ± 0.8	207 ± 4
C16:1	0.185 ± 0.004	0.73 ± 0.01	3.52 ± 0.07
C17:0	0.186 ± 0.004	0.73 ± 0.02	3.54 ± 0.08
C18:0	4.0 ± 0.1	15.7 ± 0.4	76 ± 2
C18:1*n*-9c	11.0 ± 0.1	43.3 ± 0.5	209 ± 2
C18:2*n*-6c	70 ± 1	274 ± 6	1324 ± 27
C18:3*n*-3	1.53 ± 0.05	6.0 ± 0.2	29.2 ± 0.9
C20:0	0.590 ± 0.007	2.32 ± 0.03	11.2 ± 0.1
C20:1*n*-9	0.132 ± 0.003	0.52 ± 0.01	2.50 ± 0.05
C20:3*n*-6	0.184 ± 0.004	0.72 ± 0.01	3.49 ± 0.05
C21:0	0.186 ± 0.003	0.73 ± 0.02	3.54 ± 0.08
C22:0	0.362 ± 0.006	1.42 ± 0.02	6.9 ± 0.1
C23:0	0.116 ± 0.003	0.46 ± 0.01	2.21 ± 0.05
SFA	17.4 ± 0.2	68 ± 1	331 ± 4
MUFA	11.3 ± 0.1	44.5 ± 0.5	215 ± 1
PUFA	71 ± 1	280 ± 5	1357 ± 26

The results are reported as mean ± standard deviation. C10:0—capric acid, C12:0—lauric acid, C14:0—myristic acid, C15:0—pentadecanoic acid, C16:0—palmitic acid, C16:1—palmitoleic acid, C17:0—heptadecanoic acid, C18:0—stearic acid, C18:1*n*-9c—oleic acid, C18:2*n*-6c—linoleic acid, C18:3*n*-3—α-linolenic acid, C20:0—arachidic acid, C20:1*n*-9—*cis*-11-eicosenoic acid, C21:0—heneicosanoic acid, C20:3*n*-6—*cis*-8,11,14-eicosatrienoic acid, C22:0—behenic acid, C23:0—tricosanoic acid, SFA—saturated fatty acids, MUFA—monounsaturated fatty acids, PUFA—polyunsaturated fatty acids. * Content estimated based on the conversion factor (0.8) proposed by Greenfield and Southgate [31].

**Table 4:** Tocopherol composition of goldenberry fruit and freeze-dried powder.

Constituents	Content (per 100 g)	
	Fresh Fruit	Freeze-Dried Powder
α-Tocopherol (mg)	0.579 ± 0.008	2.80 ± 0.04
β-Tocopherol (mg)	0.594 ± 0.008	2.87 ± 0.04
γ-Tocopherol (mg)	0.86 ± 0.02	4.15 ± 0.09
δ-Tocopherol (mg)	0.309 ± 0.007	1.50 ± 0.04
Σ Tocopherols (mg)	2.34 ± 0.03	11.3 ± 0.1

The results are reported as mean ± standard deviation.

**Table 5:** Phenolic and steroidal compounds tentatively identified and quantified in goldenberry fruit and calyx extracts. The retention time (Rt), wavelength of maximum absorption in the UV-vis region (λ_max_), and deprotonated and MS^2^ fragment molecules (with relative abundance in brackets) are presented.

Peak	Rt (min)	λ_max_ (nm)	[M − H]^−^ (*m*/*z*)	MS^2^ (*m*/*z*)	Tentative Identification	Reference/Type of Identification	Content (mg/g Extract)	
Fruit Extract							Hydroethanolic	Decocted
1	5.29	284	619	573 (100)	Daturametelin N	[65]	nq	-
2	5.49	278	619	573 (100)	Daturametelin N	[65]	nq	-
3	9.20	296	575	543 (100), 529 (23), 253 (5)	3-Methoxy-7-hydroxy-6-deoxyphysalin D	[66]	nq	-
4	13.50	284	557	471 (100), 495 (10), 323 (7), 121 (5)	3-Methoxyphysalin A	[66]	nq	-
5	16.66	222, 281	509	463 (100), 491 (20)	4,7-Didehydroneophysalin B	[66]	nq	-
6	18.11	233, 284	543	497 (100), 499 (12), 453 (5)	Physalin E	[66]	nq	-
7	18.59	236, 281	539	511 (100), 354 (38), 495 (5)	3-Methoxy-6,7,9,10-tetradehydrophysalin B	[66]	nq	-
**Calyx Extract**								
8	4.34	324	353	191 (100), 179 (46), 173 (18)	1-*O*-Caffeoylquinic acid	[67,68]	4.37 ± 0.09	3.22 ± 0.05
9	6.20	247, 327	353	191 (100), 179 (41), 135 (5), 173 (2)	*trans*-5-*O*-Caffeoylquinic acid	[67,68]	4.8 ± 0.1	3.94 ± 0.06
10	8.63	265, 289, 330	353	191 (100), 179 (23), 173 (8)	*cis*-5-*O*-Caffeoylquinic acid	[67,68]	1.64 ± 0.02	1.34 ± 0.02
11	16.55	256, 354	609	301 (100)	Quercetin-deoxyhexosyl-hexoside	MS/DAD	17.0 ± 0.3	10.4 ± 0.1
12	17.63	256, 352	609	301 (100)	Quercetin-deoxyhexosyl-hexoside	MS/DAD	4.3 ± 0.2	3.2 ± 0.1
13	18.58	255, 352	609	301 (100)	Quercetin-3-*O*-rutinoside (rutin)	MS/DAD	5.9 ± 0.1	3.83 ± 0.08
14	19.66	264, 348	609	301 (100), 300 (50), 302 (15)	Quercetin-deoxyhexosyl-hexoside	MS/DAD	4.0 ± 0.1	2.35 ± 0.06
					Σ Phenolic acids		10.84 ± 0.05	8.5 ± 0.1
					Σ Flavonoids		31.2 ± 0.1	19.7 ± 0.1
					Σ Phenolic compounds		42.0 ± 0.2	28.2 ± 0.1

The quantification results are presented as mean ± standard deviation, and the samples in each line differed significantly (*p* < 0.05) according to a Student’s *t*-test. Standards used in the quantification: chlorogenic acid and quercetin-3-*O*-rutinoside (the calibration curves are presented in Table S3). nq—not quantified.

**Table 6:** Bioactive properties of goldenberry fruit and calyx extracts and positive controls.

Bioactivity		Fruit Extract	Calyx Extracts		Positive Control
		Hydroethanolic	Hydroethanolic	Decocted	
**TBARS formation inhibition**					Trolox
EC_50_ values (µg/mL)		3475 ± 63 ^d^	98 ± 2 ^c^	84 ± 1 ^b^	5.4 ± 0.3 ^a^
**Oxidative hemolysis inhibition**					Trolox
IC_50_ values (µg/mL)	Δt 60 min	594 ± 10 ^d^	42 ± 1 ^b^	60 ± 1 ^c^	19.6 ± 0.7 ^a^
	Δt 120 min	880 ± 15 ^d^	86 ± 1 ^b^	115 ± 4 ^c^	41 ± 1 ^a^
	Δt 180 min	1166 ± 19 ^d^	145 ± 2 ^b^	170 ± 7 ^c^	63 ± 1 ^a^
**α-Glucosidase inhibition**					Acarbose
IC_50_ values (µg/mL)		2548 ± 623 ^c^	780 ± 157 ^b^	784 ± 226 ^b^	380 ± 19 ^a^
**AGEs formation inhibition**					Aminoguanidine
IC_50_ values (µg/mL)		na	6 ± 1 ^a^	70 ± 3 ^b^	74 ± 16 ^b^
**Lipase inhibition**					Orlistat
IC_50_ values (µg/mL)		2201 ± 511 ^c^	2055 ± 489 ^c^	1288 ± 325 ^b^	40 ± 10 ^a^
**NO production inhibition**					Dexamethasone
EC_50_ values (µg/mL)		>400 ^d^	40 ± 2 ^c^	32 ± 3 ^b^	6 ± 1 ^a^
**Cell growth inhibition**					Ellipticine
GI_50_ values (µg/mL)	AGS	>400 ^d^	170 ± 8 ^c^	22 ± 2 ^b^	0.9 ± 0.1 ^a^
	Caco-2	48 ± 3 ^d^	20 ± 2 ^c^	14 ± 1 ^b^	0.8 ± 0.1 ^a^
	MCF-7	>400 ^c^	73 ± 5 ^b^	66 ± 6 ^b^	1.020 ± 0.004 ^a^
	NCI-H460	127 ± 4 ^c^	14.2 ± 0.5 ^b^	13 ± 1 ^b^	1.01 ± 0.01 ^a^
	PLP2	>400 ^d^	72 ± 5 ^c^	37 ± 3 ^b^	1.4 ± 0.1 ^a^

The results are presented as mean ± standard deviation. In each line, different letters (a–d) indicate statistically significant differences (*p* < 0.05) between samples according to the one-way ANOVA. AGS—gastric adenocarcinoma; Caco-2—colorectal adenocarcinoma; MCF-7—breast adenocarcinoma; NCI-H460—non-small-cell lung carcinoma; PLP2—porcine liver primary cell culture. na—no inhibitory activity was observed during screening at 1 mg/mL.

**Table 7:** Antimicrobial activity of goldenberry fruit and calyx extracts and positive controls.

Microorganisms	Fruit Extract		Calyx Extracts				Positive Controls			
	Hydroethanolic		Hydroethanolic		Decocted		Sodium Benzoate		Potassium Metabisulfite	
Bacterial Strains	MIC	MBC	MIC	MBC	MIC	MBC	MIC	MBC	MIC	MBC
*Staphylococcus aureus*	1.57	3.14	0.75	1.51	1.50	3.00	4.00	4.00	1.00	1.00
*Bacillus cereus*	0.79	1.57	0.38	0.75	0.38	0.75	0.50	0.50	2.00	4.00
*Listeria monocytogenes*	1.57	3.14	1.51	3.01	1.50	1.50	1.00	2.00	0.50	1.00
*Salmonella enterica* subsp. *enterica* ser. Typhimurium	1.57	3.14	1.51	3.01	0.75	1.50	1.00	2.00	1.00	1.00
*Escherichia coli*	1.57	3.14	0.75	1.51	1.50	3.00	1.00	2.00	0.50	1.00
*Enterobacter cloacae*	3.14	6.28	1.51	3.01	1.50	3.00	2.00	4.00	0.50	0.50
Fungal Strains	MIC	MFC	MIC	MBC	MIC	MBC	MIC	MFC	MIC	MFC
*Aspergillus fumigatus*	0.79	1.57	1.51	3.01	0.75	1.50	1.00	2.00	1.00	1.00
*Aspergillus versicolor*	0.39	0.79	0.38	0.75	0.38	0.75	2.00	4.00	1.00	1.00
*Aspergillus niger*	0.79	1.57	0.75	1.51	0.75	1.50	1.00	2.00	1.00	1.00
*Penicillium funiculosum*	0.79	1.57	0.75	1.51	0.75	1.50	1.00	2.00	0.50	0.50
*Penicillium verrucosum* var. *cyclopium*	0.79	1.57	0.75	1.51	1.50	3.00	2.00	4.00	1.00	1.00
*Trichoderma viride*	0.79	1.57	0.75	1.51	1.50	3.00	1.00	2.00	0.50	0.50

MIC—minimum inhibitory concentration (mg/mL), MBC—minimum bactericidal concentration (mg/mL), MFC—minimum fungicidal concentration (mg/mL). The 30% ethanol used as a negative control did not affect microbial growth at the highest concentration (40 µL of 30% ethanol/100 µL).

## Data Availability

Data are contained within the article and Supplementary Materials.

## Supplementary Materials

The following supporting information can be downloaded at: https://www.mdpi.com/article/10.3390/plants14030327/s1, Figure S1. HPLC-chromatographic profile of phenolic and steroidal compounds in *P. peruviana* fruit extract recorded at (a) 280 nm and (b) 370 nm, and in calyx hydroethanolic extract recorded at (c) 330 nm. See Table 5 for compound identification. Table S1: Chemicals, standards, and biological materials used in the analyses; Table S2: Equipment used in the chromatographic analysis; Table S3: Seven-level calibration curves used in the quantification of phenolic compounds.
