# Supplementary material for: Nutrients, Phytochemicals, and In Vitro Biological Activities of Goldenberry (Physalis peruviana L.) Fruit and Calyx
Source: Plants (Basel). 2025 Jan 22;14(3):327. doi: 10.3390/plants14030327 (PMC11820769; doi:10.3390/plants14030327)
Supplement: Supplementary file 1 [file plants-14-00327-s001.zip › plants-3394718-supplementary.pdf]

plants

MDPI

Supplementary Material

Table S1. Chemicals, standards, and biological materials used in the analyses.

**Chemicals, standards, biological material**	**Suppliers**
Solvents for chromatographic analysis	Merck, Darmstadt, Germany
Chlorogenic acid and quercetin-3-*O*-glucoside (purity > 98%)	Extrasynthèse, Genay, France
6-Hydroxy-2,5,7,8-tetramethylchroman-2-carboxylic acid (Trolox), 2,2'-azobis(2-methylpropionamidine) dihydrochloride (AAPH) dexamethasone, sulforhodamine B, lipopolysaccharides from *Escherichia coli* O111:B4 (LPS), ellipticine, Griess reagent system kit, sodium benzoate (E211), and potassium metabisulfite (E224)	Sigma-Aldrich, St. Louis, MO, USA
α-Glucosidase from *Saccharomyces cerevisiae*, *p*-nitrophenyl-α-D-glucopyranoside (PNPG), acarbose, aminoguanidine, lipase from porcine pancreas, and 4-nitrophenyl butyrate (NPB)	Sigma-Aldrich, Barcelona, Spain
Orlistat	TCI, Barcelona, Spain
Escherichia coli (ATCC 25922), *Enterobacter cloacae* (ATCC 35030), *Salmonella enterica* subsp. *enterica* serovar Typhimurium (ATCC 13311), *Staphylococcus aureus* (ATCC 11632), *Bacillus cereus* (food isolate), *Listeria monocytogenes* (NCTC 7973), *Aspergillus fumigatus* (ATCC 9197), *Aspergillus niger* (ATCC 6275), *Aspergillus versicolor* (ATCC 11730), *Penicillium funiculosum* (ATCC 36839), *Penicillium verrucosum* var. *cyclopium* (food isolate), and *Trichoderma viride* (IAM 5061)	Mycological Laboratory, Institute for Biological Research "Sinisa Stanković", University of Belgrade Serbia
AGS (gastric adenocarcinoma), Caco-2 (colorectal adenocarcinoma), MCF-7 (breast adenocarcinoma), and NCI-H460 (non-small cell lung carcinoma)	Leibniz Institute DSMZ, Braunschweig, Germany
K, Na, Ca, and Mg standard solutions	Chemlab Analytical, AnalytiChem, Belgium
Mn, Zn, Fe, Cu, and P standard solutions	Panreac AppliChem, ITW reagents, Barcelona

Table S2. Equipment used in the chromatographic analysis of phenolic compounds.

**Equipment**	**Suppliers**
Dionex Ultimate 3000 HPLC system	Thermo Scientific, San Jose, CA, USA
Spherisorb S3 ODS-2 C18 column (3 μm, 4.6 mm × 150 mm)	Waters, Milford, MA, USA

Table S3 Seven-level calibration curves used in the quantification of phenolic compounds.

**Standard**	**Concentration range**	**Equation**	r^2	**Limit of detection**	**Limit of quantification**
Chlorogenic acid	2.5–80 μg/mL	$y = 312503x - 199432$	0.9999	0.2 μg/mL	0.68 μg/mL
Quercetin-3-*O*-rutinoside	25–800 μg/mL	$y = 13343x + 76751$	0.9998	0.18 μg/mL	0.65 μg/mL

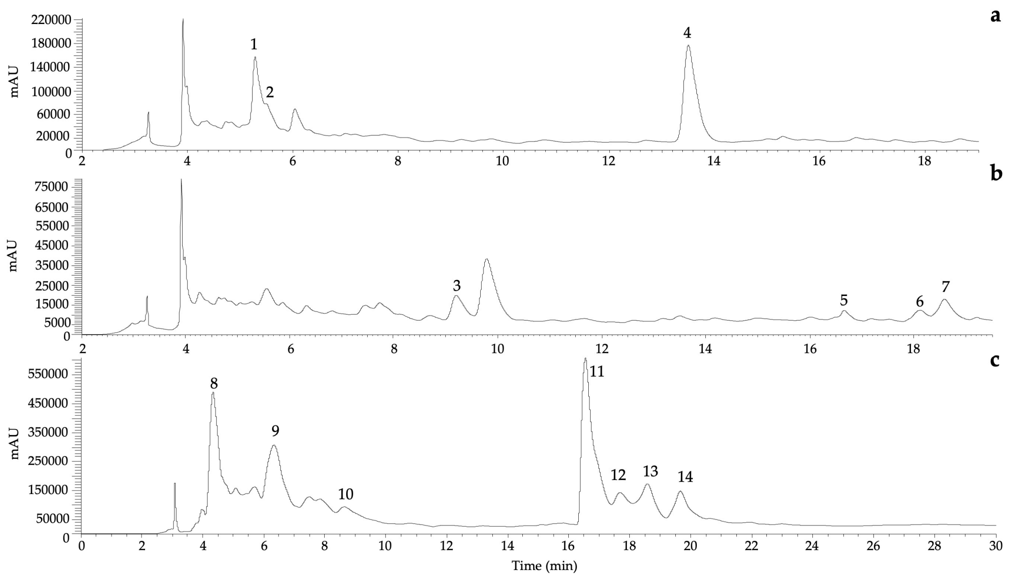

Figure S1. HPLC-chromatographic profile of phenolic and steroidal compounds in P. *peruviana* fruit extract recorded at (a) 280 nm and (b) 370 nm, and in calyx hydroethanolic extract recorded at (c) 330 nm. See Table 5 for compound identification.
